# Clinical utility of peripheral blood laboratory testing in the diagnostic workup of prurigo nodularis: A multicenter cohort study

**DOI:** 10.1016/j.jdin.2023.07.015

**Published:** 2023-08-12

**Authors:** Hannah L. Cornman, Junwen Deng, Anusha Kambala, Varsha Parthasarathy, Sriya V. Reddy, Shawn G. Kwatra

**Affiliations:** Department of Dermatology, Johns Hopkins University School of Medicine, Baltimore, Maryland

**Keywords:** diagnostic, itch, prurigo nodularis, pruritus, systemic, workup

## Abstract

**Background:**

Prurigo nodularis (PN) is a chronic inflammatory skin disease associated with several systemic comorbidities. However, there is lack of evidence supporting specific laboratory testing in the diagnostic workup of PN patients.

**Objective:**

To characterize the frequency and severity of clinical laboratory abnormalities in PN patients compared to controls.

**Methods:**

Cross-sectional study of adult patients between October, 2015 and August, 2021 using TriNetX, a global health records database encompassing over 74 million patients.

**Results:**

A total of 12,157 PN patients were matched to 12,157 controls. Significantly, greater proportions of PN patients had moderate-to-severely decreased hemoglobin, elevated transaminases, decreased albumin, increased bilirubin, increased serum creatinine, decreased estimated glomerular filtration rate, higher hemoglobin A1c levels, and alterations in thyroid stimulating hormone.

**Limitations:**

Our data identifies associated laboratory abnormalities in PN patients but is unable to support a causal relationship.

**Conclusion:**

PN patients are more likely to have laboratory abnormalities on renal, hepatic, hematologic, endocrine, and metabolic laboratory testing, demonstrating a role for systemic testing in the diagnostic workup of PN patients.

**Capsule Summary**

• There is currently a lack of clinical evidence supporting a laboratory workup of systemic conditions in prurigo nodularis (PN) patients.
• This study finds PN patients are more likely to have abnormalities in renal, hepatic, hematologic, endocrine, and metabolic laboratory testing, suggesting a role for this testing in PN diagnostic workup.

## Introduction

Prurigo nodularis (PN) is an intensely pruritic, chronic inflammatory skin disease characterized by symmetrically distributed firm nodules on the extremities and trunk.^1^^,^^2^ PN results in a significant overall reduction in health-related quality of life (QOL) among patients, and is associated with multiple systemic comorbidities including liver disease, chronic renal failure, diabetes, cardiovascular disease, infections, and malignancies.^3^, ^4^, ^5^, ^6^, ^7^, ^8^, ^9^, ^10^ The diagnostic workup and management of patients with PN are frequently challenging and systemic laboratory testing is often recommended, particularly as the treatment for PN often requires use of systemic therapy.^11^^,^^12^ However, there is a lack of data supporting peripheral blood testing for the diagnostic workup of PN. To gain a better understanding of PN-associated alterations in peripheral blood laboratory markers and their implications in PN patients, we performed a cross-sectional study comparing the frequency and severity of peripheral blood laboratory alterations in PN patients and control patients at the time of initial clinical presentation.

## Methods

### Patient population

This multicenter cross-sectional study was conducted using TriNetX, a global research network comprised of electronic medical records from over 74 million patients in 50 health care organizations, particularly in the United States, United Kingdom, Spain, Italy, India, Malaysia, and Australia. PN patients were ≥18 years old and had at least 2 diagnoses of PN using the International Classification of Diseases, Tenth Revision (ICD-10) code L28.1, validated for the identification of prurigo nodularis,^13^ from October 1, 2015 to August 31, 2021. Control patients were also ≥18 years old, had an encounter for a medical exam without specific complaint (ICD-10 codes Z00 or Z01), and had no diagnoses of dermatitis (ICD-10 codes L20-L30). As the TriNetX database utilizes only de-identified data, our study was deemed exempt from the Johns Hopkins School of Medicine Institutional Review Board (IRB).

### Laboratory parameters assessment

The index date for each patient was defined as the date on which they were first diagnosed with the ICD-10 code L28.1 for PN patients or with the ICD-10 code Z00 or Z01 for control patients. To capture laboratory values obtained close to the same time as the initial condition diagnosis, laboratory results in PN and control patients were restricted to within 1 month after the index date. As described previously,^14^, ^15^, ^16^ in order to determine clinically meaningful abnormalities in laboratory testing, laboratory results were delineated based on the National Cancer Institute Common Terminology Criteria for Adverse Events (CTCAE) v5.0 grading system, where grades 1-4 represent mild, moderate, severe, or life-threatening consequences, respectively.^17^ The CTCAE is a well-established grading system for adverse events and in this case allows for standardized reporting of laboratory-identified dysregulation. Where the CTCAE did not specifically define laboratory gradations (eg leukocytes, neutrophils, thyrotropin) or where CTCAE grades were defined relative to upper and lower limits of normal (eg alanine aminotransferase, albumin, bilirubin), the corresponding reference values published by the American Board of Internal Medicine (ABIM) were instead applied.^18^

### Statistical analysis

PN and control patients were 1:1 propensity score matched by age, sex, race, and ethnicity using a greedy nearest neighbor matching algorithm. Baseline demographics were compared using the Student’s t-test for continuous variables and a Chi-square test for categorical variables. The proportions of patients with altered laboratory values, stratified by grade of dysregulation, were analyzed using Chi-square tests for proportions. The results from Chi-square testing were interrogated through the posthoc pairwise analysis of adjusted Z-values from each comparison, and all *P*-values were adjusted for multiple hypothesis testing using the Bonferroni correction at an a priori alpha of 0.05.^19^ Results with an adjusted *P*-value < .05 were considered significant. All statistical calculations were performed using the TriNetX platform or IBM SPSS Statistics 27.

## Results

The demographic characteristics of PN and control patient cohorts are shown in Table I. We identified 12,157 PN patients, who were matched via a propensity score algorithm to 12,157 controls. Demographics of the matched cohort included a mean age of 57.9 (±16) years, 59.9% female sex, 65.5% white, and 19.9% black or African American race.

**Table I:** Demographic characteristics of prurigo nodularis (PN) patients and controls after propensity score matching

Characteristic	Prurigo nodularis (*n* = 12,157)	Controls (*n* = 12,157)	*P*-value
Age at index, mean (SD), y	58.8 (15.4)	58.8 (15.4)	1.0
Sex, no. (%)			
Males	4977 (40.9)	4977 (40.9)	1.0
Females	7180 (59.1)	7180 (59.1)	1.0
Race, no. (%)			
Caucasian	7800 (64.2)	7800 (64.2)	1.0
African American	2613 (21.5)	2613 (21.5)	1.0
Asian	326 (2.7)	326 (2.7)	1.0
Unknown	1366 (11.2)	1366 (11.2)	1.0
Ethnicity, no. (%)			
Hispanic	775 (6.4)	775 (6.4)	1.0
Not Hispanic	9469 (77.9)	9469 (77.9)	1.0

*SD*, Standard deviation; *y*, year.

**Fig 1:**
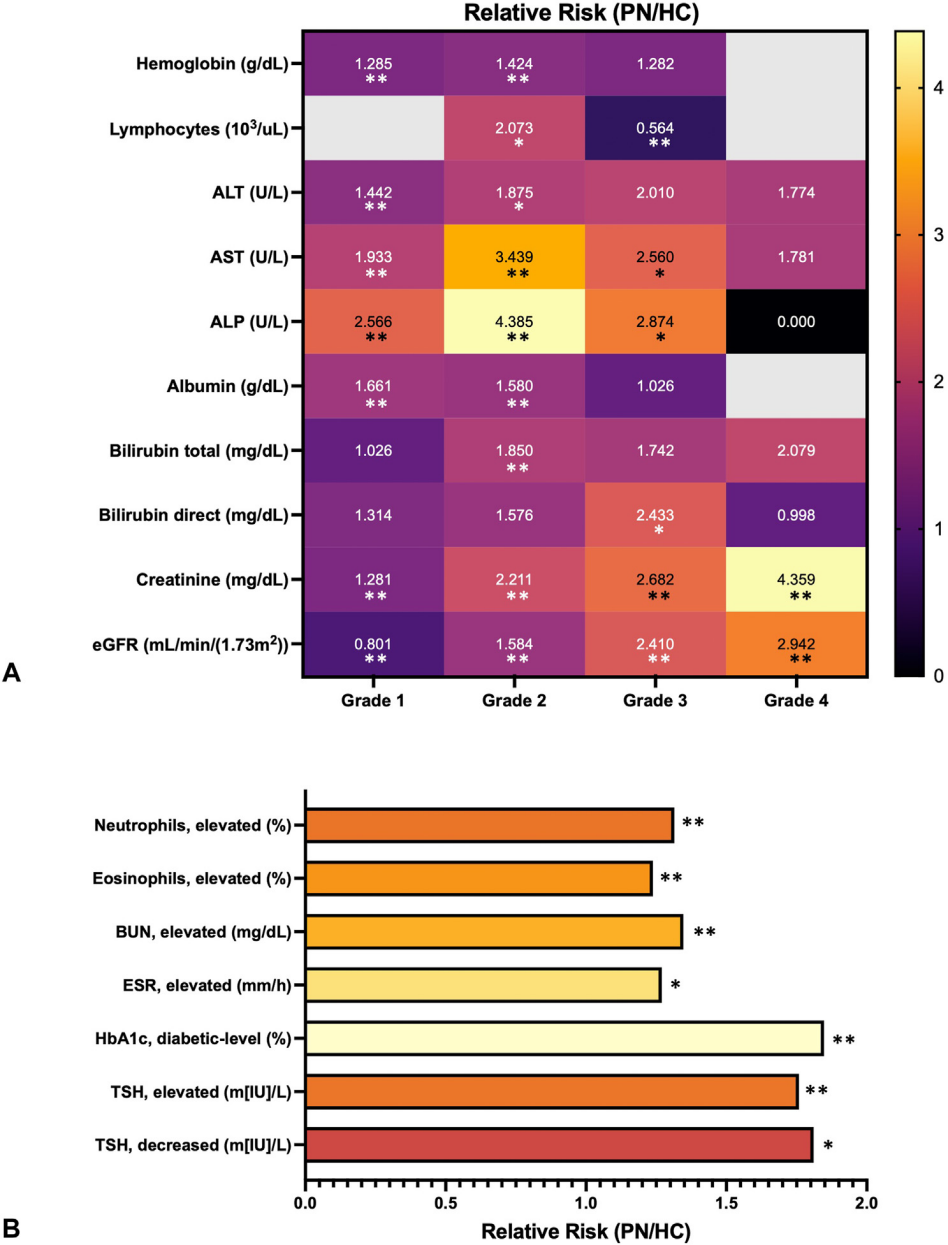
Relative risk of laboratory abnormalities in prurigo nodularis and control patients. **A,** Relative risks (*RRs*) of graded laboratory abnormalities in prurigo nodularis (*PN*) patients compared with control patients, with values of RRs depicted within each cell. **B,** RR of ungraded laboratory abnormalities in prurigo nodularis (*PN*) patients compared with control patients. ∗*P* < .05, ∗∗*P* < .0001. *ALT*, Alanine aminotransferase; *AST*, aspartate aminotransferase; *ALP*, alkaline phosphatase; *BUN*, blood urea nitrogen; *eGFR*, estimated glomerular filtration rate; *ESR*, erythrocyte sedimentation rate; *HbA1c*, hemoglobin A1c; *TSH*, thyroid stimulating hormone.

The grading criteria for defining laboratory abnormalities are shown in Supplementary Table S1, available via Mendeley at https://data.mendeley.com/datasets/hdhmvjr67z/1. Compared to controls, a significantly higher proportion of PN patients had abnormal laboratory values across several routine laboratory parameters (Table II, Table III, Table IV, Fig 1). PN patients had higher proportions of grade 2 decreases in hemoglobin (13.1% vs 9.2%, *P*-adj < .0001), grade 2 increases in lymphocytes (3.9% vs 1.9%, *P*-adj = .002), and overall ungraded elevations in neutrophils (32.4% vs 24.6%, *P*-adj < .0001) and eosinophils (43.4% vs 35.0%, *P*-adj < .0001) (Table II).

**Table II:** Hematologic laboratory abnormalities in prurigo nodularis (PN) and control patients by CTCAE grade or reference range cutoff

Parameter	Grade or cutoff parameter	PN, *n* (%)	Controls, *n* (%)	Relative risk (95% CI)	*P*-value	Adjusted *P*-value∗
Hemoglobin (g/dL)	Normal	939 (40.2)	2747 (54.4)	0.74 (0.69-0.78)	<.0001	<.0001
	Grade 1 (10.0 to < LLN)	979 (41.9)	1644 (32.6)	1.29 (1.21-1.37)	<.0001	<.0001
	Grade 2 (8.0 to <10.0)	306 (13.1)	464 (9.2)	1.42 (1.24-1.63)	<.0001	<.0001
	Grade 3 (<8.0)	114 (4.9)	192 (3.8)	1.28 (1.02-1.61)	.032	.126
Leukocytes (10^3^/uL)	Normal	1484 (84.1)	3118 (85.5)	0.98 (0.96-1.01)	.185	.370
	Elevated (>11)	280 (15.9)	529 (14.5)	1.09 (0.96-1.25)		
Lymphocytes(10^3^/uL)	Normal	1024 (89.4)	1729 (86.2)	1.04 (1.01-1.06)	.010	.041
	Grade 1 (N/A)	-	-	-	-	-
	Grade 2 (>4-20)	45 (3.9)	38 (1.9)	2.07 (1.35-3.17)	.001	.002
	Grade 3 (>20)	77 (6.7)	239 (11.9)	0.56 (0.44-0.72)	<.0001	<.0001
Neutrophils (%)	Normal	641 (67.6)	1221 (75.4)	0.90 (0.85-0.95)	<.0001	<.0001
	Elevated (>70%)	307 (32.4)	399 (24.6)	1.31 (1.16-1.49)		
Eosinophils (%)	Normal	952 (56.6)	1944 (65.0)	0.87 (0.83-0.92)	<.0001	<.0001
	Elevated (>3%)	730 (43.4)	1049 (35.0)	1.24 (1.15-1.33)		

*CI*, Confidence interval; *LLN*, lower limit of normal; *PN*, prurigo nodularis.

∗ *P*-values were adjusted using Bonferroni correction.

**Table III:** Hepatic laboratory abnormalities in prurigo nodularis (PN) and control patients by Common Terminology Criteria for Adverse Events (CTCAE) grade or reference range cutoff

Parameter	Grade or cutoff parameter	PN, *n* (%)	Controls, *n* (%)	Relative risk (95% CI)	*P*-value	Adjusted *P*-value∗
ALT (U/L)	Normal	1259 (78.6)	2438 (85.8)	0.92 (0.89-0.94)	<.0001	<.0001
	Grade 1 (>ULN-3× ULN)	278 (17.4)	342 (12.0)	1.44 (1.25-1.67)	<.0001	<.0001
	Grade 2 (>3-5× ULN)	37 (2.3)	35 (1.2)	1.88 (1.19-2.96)	.006	.032
	Grade 3 (>5-20× ULN)	17 (1.1)	15 (0.5)	2.01 (1.01-4.01)	.043	.217
	Grade 4 (>20×ULN)	10 (0.6)	10 (0.4)	1.77 (0.74-4.25)	.194	.968
AST (U/L)	Normal	1333 (78.4)	2717 (89.7)	0.87 (0.85-0.90)	<.0001	<.0001
	Grade 1 (>ULN-3× ULN)	279 (16.4)	257 (8.5)	1.93 (1.65-2.27)	<.0001	<.0001
	Grade 2 (>3-5× ULN)	56 (3.3)	29 (1.0)	3.44 (2.20-5.36)	<.0001	<.0001
	Grade 3 (>5-20× ULN)	23 (1.4)	16 (0.5)	2.56 (1.36-4.83)	.003	.013
	Grade 4 (>20× ULN)	10 (0.6)	10 (0.3)	1.78 (0.74-4.27)	.190	.951
ALP (U/L)	Normal	3194 (91.1)	1311 (76.8)	0.84 (0.82-0.87)	<.0001	<.0001
	Grade 1 (>ULN-2.5× ULN)	335 (19.6)	268 (7.6)	2.57 (2.21-2.9)	<.0001	<.0001
	Grade 2 (>2.5-5× ULN)	47 (2.8)	22 (0.6)	4.39 (2.65-7.25)	<.0001	<.0001
	Grade 3 (>5-20× ULN)	14 (0.8)	10 (0.3)	2.87 (1.28-6.46)	.007	.037
	Grade 4 (>20×ULN)	0 (0.0)	10 (0.3)	0.00	.027	.136
Albumin (g/dL)	Normal	1308 (71.7)	2892 (81.3)	0.88 (0.85-0.91)	<.0001	<.0001
	Grade 1 (3-LLN g/dL)	291 (16.0)	342 (9.6)	1.66 (1.44-1.92)	<.0001	<.0001
	Grade 2 (2-<3 g/dL)	153 (8.4)	189 (5.3)	1.58 (1.29-1.94)	<.0001	<.0001
	Grade 3 (<2 g/dL)	71 (3.9)	135 (3.8)	1.03 (0.77-1.36)	.857	1
Bilirubin, total (mg/dL)	Normal	1238 (81.9)	2759 (86.1)	0.95 (0.93-0.98)	.0002	.001
	Grade 1 (>ULN-1.5xULN)	151 (10.0)	306 (9.7)	1.03 (0.85-1.23)	.631	1
	Grade 2 (>1.5-3× ULN)	81 (5.4)	91 (2.9)	1.85 (1.38-2.48)	<.0001	.0001
	Grade 3 (>3-10× ULN)	31 (2.1)	37 (1.2)	1.74 (1.38-2.48)	.016	.080
	Grade 4 (>10× ULN)	10 (0.7)	10 (0.3)	2.08 (0.87-4.98)	.085	.427
Bilirubin, direct (mg/dL)	Normal	173 (52.7)	246 (68.9)	0.76 (0.68-0.87)	<.0001	<.0001
	Grade 1 (>ULN-1.5× ULN)	64 (19.5)	53 (14.8)	1.31 (0.94-1.83)	.105	.526
	Grade 2 (>1.5-3× ULN)	42 (12.8)	29 (8.1)	1.58 (1.01-2.47)	.044	.222
	Grade 3 (>3-10× ULN)	38 (11.6)	17 (4.8)	2.43 (1.40-4.22)	.001	.005
	Grade 4 (>10× ULN)	11 (3.4)	12 (3.4)	1.00 (0.45-2.23)	.992	1

*ALP*, Alkaline phosphatase; *ALT*, alanine aminotransferase; *AST*, aspartate aminotransferase; *CI*, confidence interval; *LLN*, lower limit of normal; *PN*, prurigo nodularis; *ULN*, upper limit of normal.

∗ *P*-values were adjusted using Bonferroni correction.

**Table IV:** Renal/Endocrine laboratory abnormalities in prurigo nodularis (PN) and control patients by Common Terminology Criteria for Adverse Events (CTCAE) grade or reference range cutoff

Parameter	Grade or cutoff parameter	PN, *n* (%)	Controls, *n* (%)	Relative risk (95% CI)	*P*-value	Adjusted *P*-value∗
BUN (mg/dL)	Normal	1335 (65.1)	3134 (74.1)	0.88 (0.85-0.91)	<.0001	<.0001
	Elevated (>20)	715 (34.9)	1095 (25.9)	1.35 (1.25-1.46)		
Creatinine (mg/dL)	Normal	1692 (67.3)	4269 (80.6)	0.83 (0.81-0.86)	<.0001	<.0001
	Grade 1 (>ULN-1.5× ULN)	436 (17.3)	717 (13.5)	1.28 (1.15-1.43)	<.0001	<.0001
	Grade 2 (>1.5-3× ULN)	190 (7.6)	181 (3.4)	2.21 (1.81-2.69)	<.0001	<.0001
	Grade 3 (>3-6× ULN)	107 (4.3)	84 (1.6)	2.68 (2.02-3.56)	<.0001	<.0001
	Grade 4 (>6× ULN)	89 (3.5)	43 (0.8)	4.36 (3.04-6.25)	<.0001	<.0001
eGFR (mL/min/[1.73 m^2^])	Normal	697 (29.9)	1660 (35.8)	0.84 (0.78-0.90)	<.0001	<.0001
	Grade 1 (60 to < LLN)	864 (37.0)	2147 (46.2)	0.80 (0.75-0.85)	<.0001	<.0001
	Grade 2 (30-59)	508 (21.8)	638 (13.7)	1.58 (1.43-1.76)	<.0001	<.0001
	Grade 3 (15-29)	126 (5.4)	104 (2.2)	2.41 (1.87-3.11)	<.0001	<.0001
	Grade 4 (<15)	139 (6.0)	94 (2.0)	2.94 (2.28-3.80)	<.0001	<.0001
ESR (mm/h)	Normal	85 (43.1)	95 (55.2)	0.78 (0.64-0.96)	.020	.041
	Elevated (>20)	112 (56.9)	77 (44.8)	1.27 (1.03-1.56)		
HbA1c (%)	Normal	175 (24.3)	724 (41.3)	0.59 (0.51-0.68)	<.0001	<.0001
	Prediabetes (5.7-<6.5%)	203 (28.2)	579 (33.0)	0.85 (0.75-0.98)	.019	.058
	Diabetes (>6.5%)	342 (47.5)	451 (25.7)	1.85 (1.65-2.06)	<.0001	<.0001
TSH (m [IU]/L)	Normal	499 (74.9)	1644 (85.9)	0.87 (0.83-0.91)	<.0001	<.0001
	Elevated (>4)	104 (15.6)	170 (8.9)	1.76 (1.40-2.21)	<.0001	<.0001
	Decreased (<0.5)	63 (9.5)	100 (5.2)	1.81 (1.34-2.45)	.0001	.0003

*BUN*, Blood urea nitrogen; *CI*, confidence interval; *eGFR*, estimated glomerular filtration rate; *ESR*, erythrocyte sedimentation rate; *HbA1c*, hemoglobin A1c; *LLN*, lower limit of normal; *PN*, prurigo nodularis; *TSH*, thyroid stimulating hormone; *ULN*, upper limit of normal.

∗ *P*-values were adjusted using Bonferroni correction.

Greater proportions of PN patients also had heightened liver enzyme markers and alterations in other hepatically produced markers such as albumin and bilirubin (Table III). Specifically, PN patients had grade 2 elevations in alanine aminotransferase (ALT) (2.3% vs 1.2%, *P*-adj = .032), grade 2 (3.3% vs 1.0%, *P*-adj < .0001) and grade 3 (1.4% vs 0.5%, *P*-adj = .013) elevations in aspartate aminotransferase (AST), and grade 2 (2.8% vs 0.6%, *P*-adj < .0001) and grade 3 (0.8% vs 0.3%, *P*-adj = .037) elevations in alkaline phosphatase (ALP). PN patients were also more likely to have decreases in albumin (grade 2, 8.4% vs 5.3%, *P*-adj < .0001), as well as increases in total bilirubin (grade 2, 5.4% vs 2.9%, *P*-adj = .0001) and direct bilirubin (grade 3, 11.6% vs 4.8%, *P*-adj = .005).

PN patients also had higher proportions of renal and endocrine laboratory abnormalities (Table IV). PN patients had signs of worse renal function, as higher proportions of PN patients experienced higher blood urea nitrogen (34.9% vs 25.9%, *P*-adj < .0001), any graded level of elevation in serum creatinine (*P*-adj < .0001 in all grades), and decreases in grades 2-4 of the estimated glomerular filtration rate (eGFR) (*P*-adj < .0001 in all grades). PN patients also had signs of endocrine dysfunction, as significantly higher proportions of patients had diabetic-level hemoglobin A1c (HbA1c) (47.5% vs 25.7%, *P*-adj < .0001) and increases (15.6% vs 8.9%, *P*-adj < .0001) or decreases (9.5% vs 5.2%, *P*-adj = .0003) in thyroid stimulating hormone (TSH). Finally, higher proportions of PN patients had elevated erythrocyte sedimentation rate (ESR) (56.9% vs 44.8%, *P*-adj = .041).

## Discussion

This large multicenter study on PN patients identifies laboratory abnormalities using real-world data, providing deeper insight into the diagnostic workup and management of PN. The results of our investigation demonstrate that compared to controls, PN patients have significantly higher frequencies of alterations in common laboratory tests such as complete blood count, basal metabolic panel, hepatic and renal function testing, thyroid function testing, and diabetes testing. These results reinforce the presence of systemic dysregulation in multiple axes, including endocrine, immune, hepatic, renal, and hemostatic among PN patients.

Importantly, we found that laboratory testing within 1 month of PN diagnosis identified a higher prevalence of renal insufficiency, liver dysfunction, and diabetes compared to ICD-10 code diagnoses of acute and chronic kidney disease (ICD-10 N17-19), liver disease (ICD-10 K70-77), and diabetes (ICD-10 E08-E13), respectively (*P* < .0001 for all). For example, 25.2% of PN patients had a prior comorbid diagnosis of acute or chronic kidney disease, whereas 33.2% of PN patients had CTCAE grade 2-4 abnormalities in eGFR, which indicates renal dysfunction significant enough to recommend intervention.^17^ Additionally, we found that 20.3% of PN patients had a prior diagnosis of liver disease but 27.8% had CTCAE Grade 2-4 abnormalities in direct bilirubin. Finally, we found that 31.6% of PN patients had a prior diagnosis of diabetes, but 47.5% of PN patients had a diabetic range HbA1c (greater than 6.5%). These findings suggest that laboratory testing has potential to identify more patients with comorbidities than medical record review alone, and is therefore beneficial for patient management.

This study not only adds to the existing body of literature demonstrating an increased prevalence of comorbid systemic conditions in patients with PN, but it also quantitatively characterizes laboratory abnormalities present in PN patients, which to our knowledge, has not yet been reported on in the literature. The severity of laboratory abnormalities we revealed underscores the severity of comorbid disease in this population and has significant implications for treatment selection and management of the overall health of PN patients.

Specifically, we found that patients with PN have significantly increased odds of CTCAE grade 3 AST/ALP elevation, direct bilirubin elevation, creatinine elevation, and eGFR reduction. CTCAE grade 3 laboratory abnormalities are considered to be severe or medically significant, disabling, and an indication for hospitalization or prolongation of hospitalization.^17^ Additionally, PN patients have significantly increased odds of CTCAE grade 4 laboratory abnormalities in creatinine and eGFR. CTCAE Grade 4 laboratory abnormalities are considered to have life-threatening consequences and urgent intervention is indicated.^17^

Regarding treatment selection, numerous medications which are commonly used to treat PN require dose adjustment or are contraindicated based on liver or renal function impairment. For example, guidelines recommend dose reduction of gabapentin and pregabalin for patients with CTCAE grades 2-4 abnormalities in eGFR,^20^^,^^21^ all of which we found were more likely to be present in PN patients. Additionally, guidelines recommend dose reduction of methotrexate for patients with CTCAE grade 1 abnormalities in eGFR, avoidance of methotrexate use in most patients with CTCAE grades 2-4 abnormalities in eGFR (eGFR <50), and dose reduction in patients with CTCAE grades 2-4 abnormalities in transaminases.^22^^,^^23^ All of these abnormalities, again, were more likely to be present in PN patients. Obtaining this information on laboratory abnormalities during the initial evaluation of a PN patient can improve the management of PN patients’ overall well-being and guide therapeutic selection.

Focusing on specific laboratory abnormalities revealed in our study, we found that significantly higher proportions of PN patients had lower hemoglobin than controls. These results corroborate previous findings describing multiple causes of anemia in PN patients, including iron deficiency anemia and anemia of chronic disease.^24^ These anemias have also been shown to be associated with other inflammatory dermatoses such as atopic dermatitis.^25^ Abnormalities in hemoglobin and red blood cell count are therefore important parameters for physicians to monitor and address.

We also found inflammatory patterns in the laboratory results of PN patients. Significantly, greater proportions of PN patients exhibited higher lymphocytes, neutrophils, and eosinophils on laboratory testing compared to controls. Immune cells in the skin release mediators such as interleukin (IL)-31, histamine, tryptase, eosinophils, prostaglandins, and neuropeptides, which can cause intense itch and inflammation.^2^ T cell populations have been implicated as key players in the pathogenesis of PN, including T-helper (Th-)2, Th17, and Th22 cells and their cytokines.^8^^,^^26^ Lesional PN skin has also been shown to display increased neutrophil and eosinophil infiltrates.^27^ Eosinophils have been shown to play a role in the cutaneous inflammation process via degranulation into the skin of PN patients, where they promote inflammation and can induce pruritus through nerve fiber modulation.^28^^,^^29^ Furthermore, ESR was also found to be significantly elevated in PN patients. ESR is a marker that is associated with a variety of systemic comorbidities, including diabetes mellitus, renal disease, heart diseases, infections, and malignancy.^30^ These results reinforce the systemic nature of inflammation in PN, and are consistent with prior studies which have found elevated levels of various inflammatory markers in the peripheral blood of PN patients.^26^^,^^31^^,^^32^ Systemic inflammation has potential to mediate greater morbidity burden in PN, highlighting the importance of complete blood count with differential testing in the workup of PN patients.

Our results also revealed severe (CTCAE grade 3 and grade 4) hepatic and renal dysregulation in PN. These significant levels of hepatic and renal dysfunction should be addressed in collaboration with the appropriate specialists or primary care providers, both to improve the overall well-being of PN patients, and because they are known to cause pruritus and could therefore be contributing factors to the development of PN. Compared to controls, higher proportions of PN patients had elevations in AST, ALT, ALP, and total and direct bilirubin. PN patients should be evaluated as clinically indicated for underlying etiologies, including cholestatic hepatitis and viral hepatitis, which have been previously reported in association with PN.^4^^,^^33^ PN patients also tended to have significantly decreased levels of albumin, which can be caused by hepatic underproduction or increased renal losses. Additionally, higher proportions of PN patients had elevated blood urea nitrogen (BUN) and creatinine levels and lower eGFR than controls. Renal disease has been documented in association with PN, and end stage renal disease is one of the top reasons for hospital admission in PN patients.^6^ Since African Americans are disproportionately affected by PN, and African American patients with PN tend to have faster progression of chronic renal disease and higher comorbidity burden compared to their Caucasian counterparts, screening for renal disease may be particularly useful in this patient population.^6^^,^^34^^,^^35^

Our results additionally corroborated prior studies that reported the association of PN with metabolic syndrome and endocrine conditions. We found that more PN patients had diabetic-grade hemoglobin A1c levels compared to controls, affirming PN’s association with diabetes.^4^^,^^10^^,^^36^ HbA1c testing can be beneficial to dermatologists during their initial assessment of PN patients for several reasons. First, diabetes is widely recognized to be associated with pruritus and neuropathy, both of which have the potential to exacerbate PN pathogenesis. Consequently, incorporating A1c testing during the initial evaluation of PN patients can facilitate a more comprehensive understanding of the underlying contributors to PN development. Moreover, this knowledge can foster collaboration with endocrine specialists or primary care providers, enabling efforts to improve glucose control. By addressing glucose control issues, dermatologists can potentially ameliorate the impact of diabetes-related pruritus, ultimately benefiting both the patient's PN condition and their overall well-being. Finally, higher proportions of PN patients also had abnormalities on thyroid function, with PN patients demonstrating significantly increased odds of having both elevated or decreased TSH. The association of prurigo nodularis with hypothyroidism has previously only been reported in a case report, and there is no published association of PN with hyperthyroidism.^37^ Thus, our results suggest that thyroid disease may be more prevalent in PN patients than previously known, and provide a basis for further investigation of comorbid thyroid disease in the PN population.

Limitations of this study include potential misclassification of PN in the electronic medical records. To counteract this issue, we required patients to have been diagnosed with PN at least twice in their health record. Because the electronic diagnosis of prurigo nodularis was only introduced along with the instatement of the ICD-10 system in October 2015, measurement of patient outcomes was limited to only 5 years. Furthermore, the exact causes of the patients’ laboratory dysregulation could not be elicited from the database.

In conclusion, PN is significantly associated with alterations in laboratory parameters on peripheral blood testing which can be detected at the patient’s initial clinical presentation. Of clinical significance, PN patients more frequently have severe laboratory abnormalities for which intervention is indicated, and which may impact therapeutic selection. Therefore, the care of PN patients should include consideration for other systemic comorbidities, and clinicians should maintain a high index of suspicion for differential diagnoses that include renal, hepatic, hematologic, endocrine, and metabolic diseases. Based on the findings of this study, the recommended initial workup for patients with PN includes a complete blood count, renal function test, liver function test, thyroid function tests, and hemoglobin A1c.

## Conflicts of interest

Dr Kwatra is an advisory board member/consultant for Abbvie, Aslan Pharmaceuticals, Arcutis Biotherapeutics, Castle Biosciences, Celldex Therapeutics, Galderma, Genzada Pharmaceuticals, Incyte Corporation, Johnson & Johnson, Leo Pharma, Novartis Pharmaceuticals Corporation, Pfizer, Regeneron Pharmaceuticals, and Sanofi and has served as an investigator for Galderma, Incyte, Pfizer, and Sanofi. All other authors report no conflicts of interest.
